# Chemical Profiling and Multimodal Anti-Inflammatory Activity of *Eugenia pyriformis* Leaves Essential Oil

**DOI:** 10.3390/molecules31020342

**Published:** 2026-01-19

**Authors:** Larissa Saviani Ribeiro, Vitor Guimarães Lourenço, Kaique Gonçalves de Souza, Yasmin Cometti Sardinha, Kevin Costa Miranda, Francisco Paiva Machado, Rômulo Augusto de Abreu Franchini, Mariana Toledo Martins Pereira, Leandro Rocha, Vinicius D’Avila Bitencourt Pascoal, Aislan Cristina Rheder Fagundes Pascoal

**Affiliations:** 1 Research Laboratory of Natural Products and Bioactive Molecules, Nova Friburgo Health Institute, Fluminense Federal University—UFF, Nova Friburgo 28625-650, RJ, Brazil; larissasaviani@id.uff.br (L.S.R.); kaiqueg@id.uff.br (K.G.d.S.); comettiyasmin@gmail.com (Y.C.S.); marianatmartinsp@gmail.com (M.T.M.P.); viniciuspascoal@id.uff.br (V.D.B.P.); 2 Laboratory of Natural Products Technology, Department of Pharmaceutical Technology, School of Pharmacy, Fluminense Federal University, Niterói 24241-000, RJ, Brazil; kevincosta.cm@gmail.com (K.C.M.); fmachado@id.uff.br (F.P.M.); lean.machado@gmail.com (L.R.); 3 Graduate Program in Sciences Applied to Health Products, Fluminense Federal University, Niterói 24241-000, RJ, Brazil; 4 Department of Basic Sciences, Nova Friburgo Health Institute, Fluminense Federal University—UFF, Nova Friburgo 28625-650, RJ, Brazil

**Keywords:** *Eugenia pyriformis*, Myrtaceae, anti-inflammatory activity, sesquiterpenes, pro-inflammatory cytokines

## Abstract

*Eugenia pyriformis* Cambess., popularly known as uvaia, is a native Brazilian species belonging to the Myrtaceae family that has attracted pharmacological interest due to its richness in bioactive secondary metabolites. Previous studies have reported antimicrobial and antioxidant activities of the essential oil obtained from its leaves, reinforcing its therapeutic potential. In this context, the present study aimed to extract and characterize the essential oil from *E. pyriformis* leaves cultivated in the mountainous region of Rio de Janeiro, Brazil, and to evaluate its anti-inflammatory potential through in vitro and in vivo models. Gas chromatography mass spectrometry (GC–MS) analysis revealed a predominance of sesquiterpene hydrocarbons, mainly γ-muurolene, δ-cadinene, and β-caryophyllene. The oil exhibited significant anti-edematogenic activity in carrageenan-, prostaglandin E_2_-, and bradykinin-induced paw edema models in adult female Swiss mice, suggesting modulation of inflammatory mediators, possibly through inhibition of the cyclooxygenase (COX) pathway. Conversely, no effect was observed in the compound 48/80-induced model, indicating the absence of activity on histamine- and serotonin-mediated processes. *In vitro* assays demonstrated that the oil reduced TNF-α and IL-1β gene expression in RAW 264.7 macrophages, confirming its ability to modulate pro-inflammatory cytokines. Taken together, these findings demonstrate that the essential oil of *E. pyriformis* exerts anti-inflammatory activity through multiple targets.

## 1. Introduction

Natural products—especially those obtained from plant species—have been central to human healthcare since ancient times, shaping the foundations of traditional medical practices across civilizations [1]. Evidence from Mesopotamian texts dated to around 2600 B.C. documents the preparation of over a thousand plant-based remedies used to manage ailments such as respiratory infections, parasitoses, and inflammatory disorders. Notably, several of these botanical resources continue to be relevant in contemporary therapeutic strategies [2], as their diverse biological properties contribute to interventions for an estimated 87% of known human diseases [3].

Within this framework, numerous natural products possess anti-inflammatory properties and have long been incorporated into traditional healing practices for managing conditions such as pain, fever, and arthritis [4]. Well-known examples include acetylsalicylic acid, originally isolated from willow species (*Salix* sp.), historically used to alleviate febrile states and pain [5,6]; colchicine, an alkaloid obtained from *Colchicum autumnale* and commonly prescribed for chronic gout; and the essential oil of *Cordia verbenacea* DC, indicated for disorders such as chronic tendinitis and myofascial pain [7,8]. Edible plants also function as nutraceuticals because they contain secondary metabolites with multiple biological activities, including anti-inflammatory effects [9,10,11]. Although inflammation represents a normal physiological response, its dysregulation can result in tissue damage and contribute to the development of diseases such as arthritis, multiple sclerosis, and cancer [12,13,14]. Moreover, many anti-inflammatory agents currently in clinical use are associated with undesirable effects, particularly during long-term therapy [15], highlighting the ongoing need for safer and more effective therapeutic alternatives.

*Eugenia pyriformis*, commonly called uvaia, is a Brazilian native species [16] that has gained increasing attention due to its rich composition of bioactive secondary metabolites. Earlier investigations reported antimicrobial and antioxidant effects associated with the essential oil obtained from its leaves, emphasizing its promise as a natural source of high-value compounds [17,18]. In this context, the present work extracted and characterized the leaf essential oil of *E. pyriformis* cultivated in the mountainous region of Rio de Janeiro and assessed its anti-inflammatory properties using both in vitro and in vivo approaches. The findings highlight the relevance of this species as a cultivated non-food, non-feed plant with noteworthy industrial potential. By demonstrating that its sesquiterpene-rich essential oil exhibits significant biological activity and may serve as a viable raw material for the development of bio-based products in pharmaceutical, cosmetic, and related sectors, this study strengthens the scientific foundation for the valorization of underutilized plant resources with industrial applicability.

## 2. Results

### 2.1. Phytochemical Profile Analysis

The hydrodistillation extraction of fresh leaves of *E. pyriformis* yielded 0.21% essential oil (*w*/*w*). Phytochemical characterization by GC-MS allowed the identification of 48 compounds, representing 86.52% of the essential oil. The analysis revealed a predominant fraction of sesquiterpene hydrocarbons (67.38%), with the major components being γ-muurolene (18.44%), δ-cadinene (9.02%), and β-caryophyllene (7.89%) (Table 1).

### 2.2. In Vitro Analysis of Anti-Inflammatory Effects

**Figure 1:**
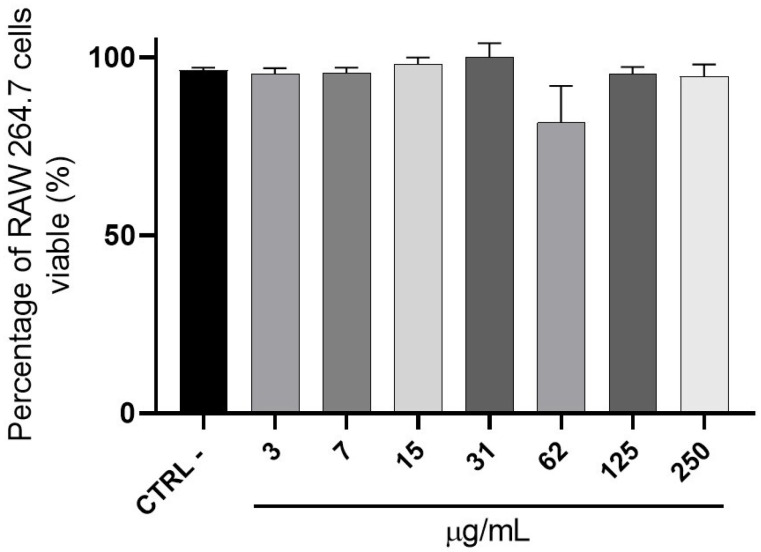
Evaluation of cell viability by the MTT assay in RAW 264.7 cells after treatment with *E. pyriformis* essential oil. Data are presented as mean ± SEM. One-way ANOVA followed by Dunnett’s multiple comparison test was used to analyze differences between the control (cells and culture medium) and treatment groups. *p* < 0.05 was considered statistically significant.

In an MTT cell viability assay using RAW cells, *E. pyriformis* essential oil at concentrations ranging from 3 μg/mL to 250 μg/mL did not significantly affect cellular metabolism compared with the control group (Figure 1). Therefore, it was not possible to determine the IC_50_ value for this sample, since at all tested concentrations, the percentage of cell viability remained above 50%.

**Figure 2:**
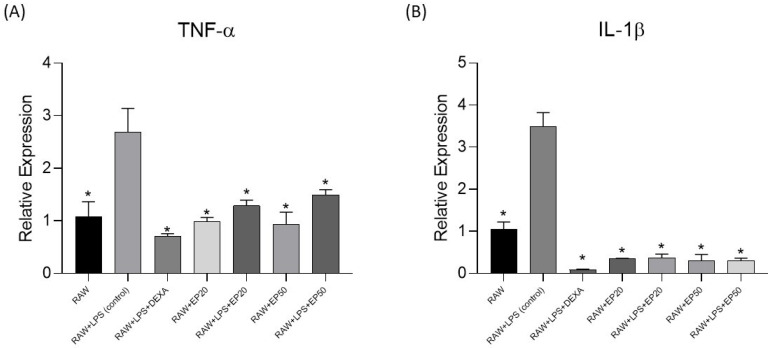
Relative expression of the pro-inflammatory genes TNF-α (**A**) and IL-1β (**B**) in RAW 264.7 cells, expressed as Fold Change values. RT-qPCR analysis showed increased gene expression after LPS stimulation and a modulatory effect of the treatments relative to the LPS group. Statistical analysis was performed using one-way ANOVA followed by Dunnett’s test with GraphPad Prism 8 software. Data are presented as mean ± standard deviation. All group comparisons to the RAW + LPS, differences were considered statistically significant when (*) *p* < 0.05.

For the evaluation of TNF-α and IL-1β gene expression by RT-PCR, RAW 264.7 cells were treated for 24 h with *E. pyriformis* essential oil at concentrations of 20 and 50 μg/mL, in the absence or presence of LPS. Cells were also treated with the anti-inflammatory drug dexamethasone (1 μM). RAW 264.7 cells stimulated with LPS served as the control. The expression levels (Figure 2) of TNF-α and IL-1β in the LPS-stimulated groups treated with dexamethasone (1 μM) and *E. pyriformis* essential oil (20 and 50 μg/mL) after 24 h were statistically different from the control group (RAW + LPS). A similar pattern was observed in groups in which RAW cells were not stimulated with LPS. Therefore, in the experimental groups, a reduction in the relative expression of the pro-inflammatory genes TNF-α and IL-1β was observed compared with the control group.

### 2.3. In Vivo Analysis of Anti-Inflammatory Effects

**Figure 3:**
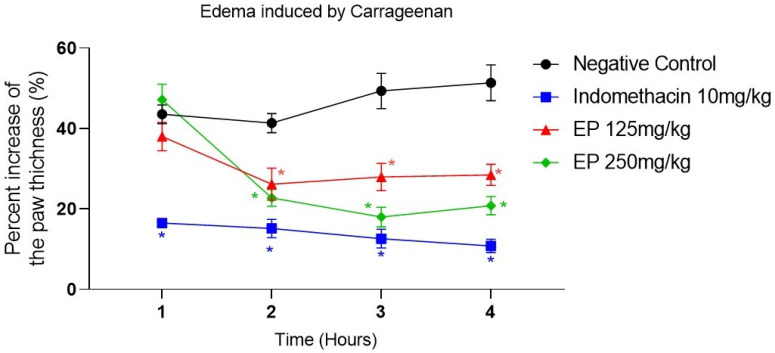
*E. pyriformis* activity in edema induced by carrageenan. The effect of EP 250 mg/kg, EP 125 mg/kg, and Indomethacin 10 mg/kg on carrageenan-induced paw edema is expressed as a percentage of increase in paw thickness (%)/hours after induction of edema, where * differed statistically (*p* < 0.05) from the saline group. Statistical analysis was performed using one-way ANOVA followed by Dunnett’s multiple comparisons test.

In the carrageenan-induced paw edema model, both doses of *E. pyriformis* essential oil showed a statistically significant difference compared with the negative control starting from the second hour after induction and remaining significant at subsequent time points (Figure 3 and Figure S1). This result indicates the sample’s ability to inhibit edema formation. The percentage of paw thickness inhibition in the experimental groups ranged from 62.00% to 71.52% in the EP 125 mg/kg group and from 52.83% to 79.17% in the EP 250 mg/kg group. The indomethacin control group (10 mg/kg) showed a statistically significant difference at all experimental time points, demonstrating its ability to suppress edema development, with paw thickness inhibition values ranging from 83.48% to 89.19%.

**Figure 4:**
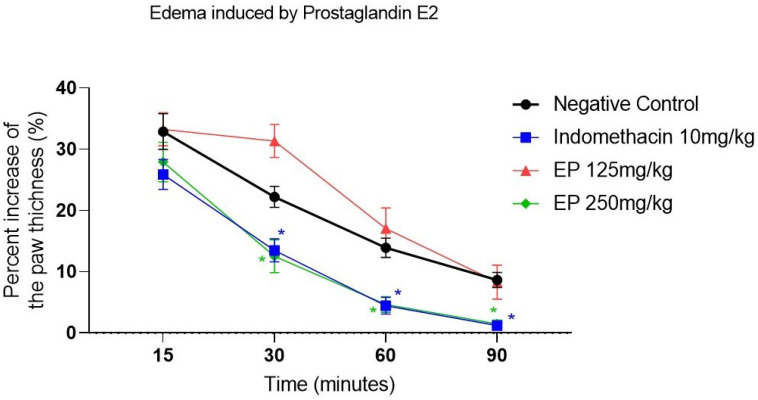
*E. pyriformis* activity in edema induced by Prostaglandin E_2_. The effect of EP 250 mg/kg, 125 mg/kg, and Indomethacin 10 mg/kg on prostaglandin E_2_-induced paw edema is expressed as a percentage of increase in paw thickness (%)/minutes after induction of edema, where * differed statistically (*p* < 0.05) from the saline group. Statistical analysis was performed using one-way ANOVA followed by Dunnett’s multiple comparisons test. *p*-values < 0.05 were considered significant.

In the prostaglandin E_2_-induced paw edema model, among the experimental groups, only *E. pyriformis* essential oil at 250 mg/kg exhibited anti-edematogenic activity, indicating its ability to inhibit edema formation (Figure 4 and Figure S2). Accordingly, EP 250 mg/kg showed a statistically significant difference compared with the negative control starting from the second point of the experiment, i.e., 30 min after induction. In this group, the percentage of paw thickness inhibition ranged from 72.09% to 98.51%. The positive control, indomethacin (10 mg/kg), showed a similar pattern, with statistically significant differences at the last three experimental time points and edema inhibition percentages ranging from 74.13% to 98.79%.

**Figure 5:**
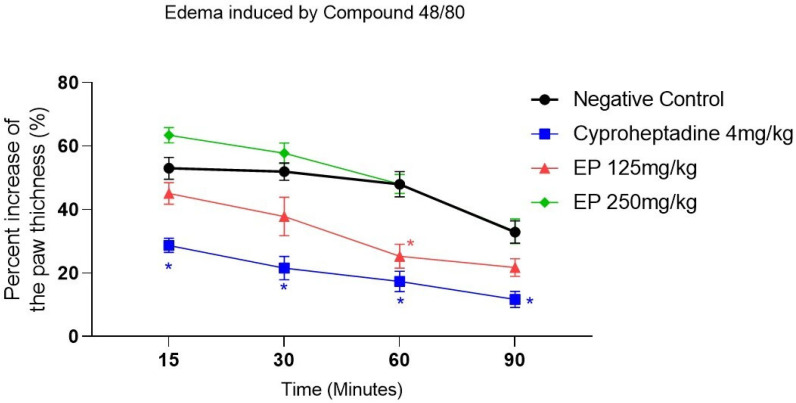
*E. pyriformis* activity in edema induced by Compound 48/80. The effect of EP 250 mg/kg, 125 mg/kg, and cyproheptadine 4 mg/kg on Compound 48/80-induced paw edema is expressed as a percentage of increase in paw thickness (%)/minutes after induction of edema, where * differed statistically (*p* < 0,05) from the saline group. Statistical analysis was performed using one-way ANOVA followed by Dunnett’s multiple comparisons test. *p*-values < 0.05 were considered significant.

In the compound 48/80-induced paw edema model, the positive control, cyproheptadine (4 mg/kg), showed a statistically significant difference compared with the negative control at all evaluated time points—15, 30, 60, and 90 min after induction (Figure 5 and Figure S3). In this group, the percentage of paw thickness inhibition ranged from 71.29% to 88.29%. In contrast, the experimental groups treated with *E. pyriformis* essential oil at both doses failed to inhibit edema formation in this model, as only the EP 125 mg/kg group showed a statistically significant difference compared with the negative control at 60 min after induction.

**Figure 6:**
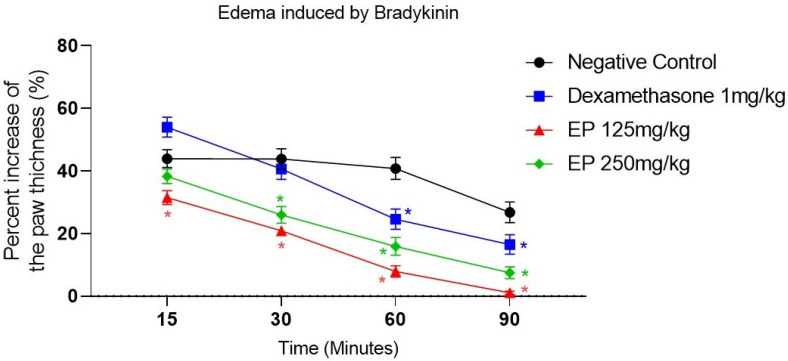
*E. pyriformis* activity in edema induced by bradykinin. The effect of EP 250 mg/kg, 125 mg/kg, and dexamethasone 1 mg/kg on bradykinin-induced paw edema is expressed as a percentage of increase in paw thickness (%)/minutes after induction of edema, where * differed statistically (*p* < 0.05) from the saline group. Statistical analysis was performed using one-way ANOVA followed by Dunnett’s multiple comparisons test. *p*-values < 0.05 were considered significant.

Finally, in the bradykinin-induced paw edema model, both doses of *E. pyriformis* essential oil exhibited anti-edematogenic activity (Figure 6 and Figure S4). The EP 125 mg/kg group showed a statistically significant difference compared with the negative control at all experimental time points, with edema inhibition percentages ranging from 68.52% to 98.9%. The EP 250 mg/kg group differed significantly from the negative control starting at the second point (30 min after induction) and remained significantly different at subsequent time points. In this group, the inhibition percentages of edema formation ranged from 61.72% to 92.49%. The positive control, dexamethasone, showed a statistically significant difference compared with the negative control at 60 and 90 min after induction, with edema inhibition percentages ranging from 46.01% to 92.49%.

## 3. Discussion

The genus *Eugenia* (Myrtaceae) comprises more than one thousand species, among which *E. uniflora* and *E. brasiliensis* stand out as producers of “pitanga” and “grumixama,” fruits widely consumed fresh or used in the preparation of juices and jams [19,20]. Species of this genus are also important sources of essential oils rich in terpenoids, particularly sesquiterpenes, which are widely applied in the pharmaceutical and cosmetic industries [19,21]. The pronounced phytochemical diversity of Eugenia species underlies their broad range of biological activities, notably their anti-inflammatory potential [19]. Phytochemical characterization of the essential oil obtained from *E. pyriformis* leaves by GC–MS revealed a predominance of sesquiterpene hydrocarbons, including γ-muurolene, δ-cadinene, and β-caryophyllene. These compounds have been extensively reported to exhibit anti-inflammatory activity in both in vivo and in vitro experimental models [22,23,24]. In general, terpenes—particularly sesquiterpene hydrocarbons—exert their anti-inflammatory effects through antioxidant mechanisms and by downregulating the expression of pro-inflammatory mediators and cytokines, such as IL-1β, IL-8, IL-10, and TNF [24,25].

Inflammation is a complex biological process involving multiple mediators and signaling pathways, which must be carefully considered when evaluating substances with potential anti-inflammatory activity. In this context, in vivo experimental models remain essential tools for elucidating the pathophysiology of inflammatory responses and for assessing the pharmacological potential of new therapeutic agents. Among the most employed models are paw edema assays induced by phlogistic agents, such as carrageenan, bradykinin, prostaglandin E_2_, and compound 48/80, each of which triggers edema formation through distinct mechanisms [26,27,28].

The carrageenan-induced paw edema model is widely used to evaluate the effects of nonsteroidal anti-inflammatory drugs (NSAIDs) and to investigate the involvement of inflammatory mediators. The early phase (up to 6 h) is characterized by the release of histamine, serotonin, bradykinin, and arachidonic acid metabolites, with histamine and serotonin predominating during the first hour, followed by bradykinin and prostaglandins between the second and third hours. The late phase (6–72 h) involves neutrophil infiltration, increased COX-2 expression, modulation of nitric oxide (NO) production, and the migration of monocytes and macrophages [29,30,31]. In this model, treatment with *E. pyriformis* essential oil (125 and 205 mg/kg) produced statistically significant inhibition of edema at 2, 3, and 4 h, suggesting an anti-edematogenic effect likely mediated by modulation of bradykinin- and prostaglandin-related pathways.

Prostaglandin E_2_, derived from arachidonic acid metabolism, plays a central role in inflammation by promoting vasodilation, leukocyte migration, mediator release, and sensitization of nociceptive nerve endings, thereby contributing to pain and edema formation [14,32]. In the prostaglandin E_2_-induced paw edema model, the essential oil of *E. pyriformis* at the highest tested dose (250 mg/kg) significantly reduced edema formation. Indomethacin, used as the positive control, inhibits COX-1 and reduces the synthesis of prostaglandins and prostacyclin, thereby reducing vasodilation and edema [15,33]. The comparable effects observed suggest that the essential oil may interfere with prostaglandin-mediated inflammatory responses.

Bradykinin is a potent vasoactive peptide generated during inflammation, resulting from increased vascular permeability and plasma extravasation. It promotes vasodilation, enhances vascular permeability, induces nitric oxide release, and modulates the production of prostaglandins and histamine [34,35]. In the bradykinin-induced paw edema model, *E. pyriformis* essential oil inhibited edema formation at both tested doses, indicating its ability to modulate bradykinin-driven inflammatory processes. Dexamethasone, used as the reference drug, exerts its effects by binding to glucocorticoid receptors and suppressing the expression of pro-inflammatory mediators, including TNF-α, IL-1β, and COX-2, as well as reducing prostaglandin and leukotriene synthesis [36,37,38]. The similar efficacy observed with the essential oil suggests a modulatory effect on the expression of pro-inflammatory mediators.

Compound 48/80 is a polybasic agent composed of low-molecular-weight polymers that induces rapid mast cell degranulation, leading to the immediate release of histamine and serotonin. It also stimulates the production of additional inflammatory mediators, including cytokines, prostaglandins, leukotrienes, and chemokines [39,40]. The essential oil of *E. pyriformis* did not exhibit significant activity in the compound 48/80-induced paw edema model. This result is consistent with the lack of effect observed during the initial phase of the carrageenan-induced edema, suggesting that the essential oil does not directly interfere with histamine- or serotonin-mediated responses.

Evaluation of TNF-α and IL-1β gene expression by RT-PCR demonstrated that *E. pyriformis* essential oil significantly reduced the expression of both cytokines in RAW 264.7 macrophages at concentrations of 20 and 50 μg/mL, regardless of LPS stimulation. Sesquiterpenes, the major chemical class identified in essential oils, are widely recognized as key contributors to the anti-inflammatory activity of essential oils from various plant species. Compounds such as β-caryophyllene, α-humulene, germacrene D, and spathulenol—commonly detected in Myrtaceae essential oils—exert anti-inflammatory effects through mechanisms involving modulation of cytokine production, inhibition of NF-κB signaling, reduction in nitric oxide levels, and attenuation of edema and leukocyte migration. Although direct evidence of cyclooxygenase inhibition by these sesquiterpenes remains limited, their ability to interfere with upstream inflammatory signaling pathways supports a multimodal anti-inflammatory profile. Furthermore, the absence of significant cytotoxicity in RAW 264.7 macrophages reinforces the preliminary safety of *E. pyriformis* essential oil and indicates that the observed effects are not secondary to reduced cell viability.

Based on the GC–MS profile, the anti-inflammatory activity of *E. pyriformis* essential oil can be mechanistically attributed to its high content of sesquiterpene hydrocarbons, particularly γ-muurolene, δ-cadinene, and β-caryophyllene. β-Caryophyllene, a significant constituent of Myrtaceae essential oils, is a well-known CB2 receptor agonist and has been reported to exert anti-inflammatory effects by inhibiting NF-κB activation, downregulating pro-inflammatory cytokines such as TNF-α and IL-1β, and modulating COX-2 and nitric oxide pathways [41]. Likewise, cadinene- and muurolene-type sesquiterpenes have been associated with reduced vascular permeability, suppression of leukocyte migration, and interference with prostaglandin- and bradykinin-mediated signaling. The predominance of these compounds supports a multimodal mechanism of action involving cytokine regulation, inhibition of inflammatory mediator synthesis, and attenuation of edema formation, which is consistent with the effects observed in the carrageenan-, prostaglandin E_2_-, and bradykinin-induced paw edema models, as well as with the reduction in TNF-α and IL-1β gene expression in macrophages.

Although the present study provides consistent evidence of the anti-inflammatory activity of *E. pyriformis* essential oil in acute experimental models, some limitations should be acknowledged. The investigation was restricted to acute inflammatory conditions, and the long-term efficacy, safety, and potential toxicity associated with repeated administration were not evaluated. Considering that chronic inflammatory diseases involve sustained activation of inflammatory pathways and complex cellular interactions, future studies should include chronic inflammation models and extended dosing regimens to better characterize the therapeutic potential and safety profile of *E. pyriformis* essential oil.

## 4. Materials and Methods

### 4.1. Essential Oil Extraction

Fresh leaves of *E. pyriformis* were collected in Nova Friburgo, Rio de Janeiro, Brazil, from a specimen cultivated in a private area. A qualified taxonomist identified the species, and an exsiccata was deposited in the Herbarium of the University of Campinas (UNICAMP) under the number UEC170007. The essential oil was extracted by hydrodistillation. Fresh plant material (360 g) was initially ground in distilled water, placed in a 5 L round-bottom flask, and subjected to hydrodistillation using a modified Clevenger apparatus. After 4 h of extraction, the obtained essential oil was stored at 4 °C in glass vials until further analysis. To obtain sufficient mass, this part of the experiment had to be repeated 4 times.

### 4.2. Phytochemical Profile Analysis

The chemical composition of the essential oil of *E. pyriformis* was determined by gas chromatography–mass spectrometry (GC-MS) using a GC-MS-QP 500 gas chromatograph (Shimadzu, Kyoto, Japan) equipped with a quadrupole mass spectrometer detector and electron impact ionization (70 eV) at a scan rate of 1 scan/s. Quantification was performed using a GC-2014 gas chromatograph (Shimadzu, Japan) with a flame ionization detector (GC-FID). The essential oil sample was dissolved in CH_2_Cl_2_ (1:100 mg/mL) and injected. Chromatographic conditions were as follows: injector temperature was set at 260 °C, helium was used as the carrier gas at a flow rate of 1 mL/min, with a split ratio of 1:40. A DB-5 column (30 m × 0.32 mm i.d. × 0.25 μm film thickness) was used for GC-MS analysis, with the oven temperature initially set at 60 °C and increased at a rate of 3 °C/min to 290 °C. The arithmetic retention index (RI) was calculated by interpolation of retention times of a standard mixture of n-alkanes (C7–C40) analyzed under the same chromatographic conditions. Chemical identification of the essential oil constituents was performed by comparing RI values and mass spectral fragmentation patterns with those in the literature [42] (Adams, 2017). Fragmentation patterns were also compared with mass spectral profiles in the National Institute of Standards and Technology (NIST) database. The relative abundance of the essential oil constituents was determined by normalizing peak areas from GC-FID analysis under the same chromatographic conditions as GC-MS, except that the detector temperature was set to 290 °C.

### 4.3. In Vitro Analysis of Anti-Inflammatory Effects

#### 4.3.1. Cell Culture

For the experiments, a murine macrophage cell line (RAW 264.7) was used. The cells were cultured in Dulbecco’s Modified Eagle Medium (DMEM) supplemented with 10% (*v*/*v*) fetal bovine serum (FBS) and 1% (*v*/*v*) penicillin–streptomycin. Cultures were incubated at 37 °C in a humidified atmosphere containing 5% CO_2_ and 95% air for 24 h.

#### 4.3.2. Colorimetric Metabolic Activity Assay Using MTT

Cell viability was assessed using the MTT colorimetric assay with the RAW 264.7 cell line. The cells were seeded in 96-well plates at a concentration of 1 × 10^4^ cells/well. After 24 h, they were treated with different concentrations of the essential oil of *E. pyriformis* and vehicle (medium), used as a negative control (n = 3). The cells were incubated for 48 h, after which the medium was removed and 100 μL of MTT solution (0.3 mg/mL; Invitrogen, Thermo Fisher, Hillsboro, OR, USA) was added. After 4 h, the MTT solution was removed, and the formazan crystals were dissolved in DMSO. The plates were read using an ELISA Epoch-Biotek reader (BioTek Instruments, Winooski, VT, USA) at λ = 570 nm. Statistical analysis of the data was performed using GraphPad Prism 6 (GraphPad Software, San Diego, CA, USA), employing one-way ANOVA followed by Dunnett’s test, with *p* < 0.05 considered statistically significant. Cells treated with vehicles alone were thought to have 100% cell viability. The experiments were carried out in triplicate.

#### 4.3.3. Evaluation of Gene Expression by RT-PCR

Murine macrophage RAW 264.7 cells were stimulated with LPS (1 μg/mL) in the presence of *E. pyriformis* essential oil at concentrations of 20 and 50 μg/mL or dexamethasone (1 μM) for 24 h. After this period, total RNA was isolated from whole-cell lysates using TRIzol reagent (Invitrogen, Carlsbad, CA, USA) according to the manufacturer’s protocol. Total RNA (2 μg) was used to synthesize first-strand cDNA with the High-Capacity cDNA Reverse Transcription Kit (Thermo Fisher) following the manufacturer’s instructions. For amplification of GAPDH, TNF-α, and IL1-β, the following primers were used: GAPDH, 5′ GTC TTC ACT ACC ATG GAG AAG G3′ (forward) and 5′ TCA TGG ATG ACC TTG GCC AG3′ (reverse); TNF-α, 5′ AGGGGAAATGA GAGACGCAA3′ (forward) and 5′ TTCCCCATCTCTTGCCA CAT3′ (reverse); IL1-β, 5′ TGC AGA GTT CCC CAA CTG GTA CAT C3′ (forward) and 5′ GTG CTG CCT AAT GTC CCC TTG AAT C3′ (reverse). PCR amplification was performed using Power SYBR Green PCR Mix (Thermo Fisher) under the following conditions: 94 °C for 5 min (1 cycle), followed by 40 cycles of 94 °C for 1 min, 56 °C for 30 s, and 72 °C for 1 min. Relative gene expression levels, normalized to the endogenous reference GAPDH, were calculated using the delta cycle threshold (ΔCt) method [43].

### 4.4. In Vivo Analysis of Anti-Inflammatory Effects

#### 4.4.1. Animals

Adult female Swiss mice (n = 128), weighing 25–30 g and aged 7–8 weeks, obtained from the Laboratory Animal Center of the Universidade Federal Fluminense (NAL-UFF), were used. Animals were maintained at the Multiuser Laboratory for Biomedical Research of the Instituto de Saúde de Nova Friburgo (ISNF-UFF) in appropriate cages (five animals per cage), with ad libitum access to commercial feed and water, under a 12 h light/dark cycle and controlled environmental conditions, including temperature (18–22 °C) and relative humidity (45–55%). Animals were acclimated for at least 7 days prior to the experiments. Animals were randomly allocated to experimental groups, and all procedures were conducted in accordance with international guidelines for the care and use of laboratory animals. All experimental procedures were approved by the Ethics Committee on Animal Experimentation of UFF (approval number 9518301121) before the start of the study.

#### 4.4.2. Paw Edema Induced Using Carrageenan

Anti-inflammatory activity was determined using the carrageenan-induced hind paw edema method [44]. Swiss mice were randomly assigned to four groups (n = 8/group). The animals in each experimental group were marked with permanent markers using predefined colors, and the boxes were labeled with the type of treatment being conducted. They fasted for 1 h and subsequently treated orally according to the experimental design: negative control (0.9% saline solution), positive control (indomethacin 10 mg/kg), and essential oil of *E. pyriformis* at doses of 125 and 250 mg/kg, administered orally (p.o.). The dose range was selected based on concentrations reported in the literature for essential oils rich in sesquiterpenes from Myrtaceae species, which typically exhibit anti-inflammatory activity at 50–300 mg/kg in rodent models without inducing acute toxicity. In addition, preliminary in vitro assays using RAW 264.7 macrophages demonstrated significant anti-inflammatory effects at non-cytotoxic concentrations, providing an initial indication of biological activity and safety. After 30 min, a solution of carrageenan (30 μg/paw; Sigma^®^, St. Louis, MI, USA) was injected into the right hind paw of each animal. Paw volumes were measured with a digital caliper before and at 1, 2, 3, and 4 h after carrageenan administration, by comparing paw thickness between control and treated groups. The group treated with 0.9% saline was considered the maximal-inflammation group, and all other treatments were compared with this group. Results were expressed as the percentage of paw thickness at the different time points for the phlogistic agent used. Statistical analysis was performed using one-way ANOVA followed by Dunnett’s multiple comparisons test. *p*-values < 0.05 were considered significant [45].

#### 4.4.3. Paw Edema Induced Using Prostaglandin E2, Bradyknin and Compound 48/80

Additional assays were conducted to assess the anti-inflammatory potential using different phlogistic agents. Swiss mice were randomly allocated into four groups (n = 8/group) according to treatment: negative control, positive control, and *E. pyriformis* essential oil at doses of 125 and 250 mg/kg, administered orally (p.o.). Thirty minutes after oral administration, inflammation was induced by intraplantar injection of the respective inflammatory mediators into the right hind paw. The inflammatory agents and corresponding positive controls included: Prostaglandin E2 (30 μg/paw) with indomethacin (10 mg/kg), compound 48/80 (10 μg/paw) with cyproheptadine (4 mg/kg) and bradykinin (50 μg/paw) with dexatemethasone (1 mg/kg) [33]. Paw volume was measured using a digital caliper immediately before inflammatory induction and at 15, 30, 60, and 90 min thereafter. Paw thickness was measured, and results were expressed as the percentage of swelling relative to baseline [45].

## 5. Conclusions

The essential oil from *E. pyriformis* leaves exhibited a chemical profile rich in sesquiterpene hydrocarbons, mainly γ-muurolene, δ-cadinene, and β-caryophyllene, compounds widely recognized for their anti-inflammatory properties. The oil showed significant anti-edematogenic activity in carrageenan-, bradykinin-, and prostaglandin E_2_-induced paw edema models, suggesting that its effect is associated with the modulation of inflammatory mediators, such as bradykinin and prostaglandins, possibly through inhibition of the cyclooxygenase (COX) pathway. Conversely, the absence of effect in the compound 48/80-induced model indicates that the oil does not interfere with histamine- or serotonin-mediated events during the early phase of inflammation.

Furthermore, the downregulation of TNF-α and IL-1β gene expression in RAW 264.7 macrophages treated with the oil corroborates its in vitro anti-inflammatory potential, consistent with the activity observed in vivo.

Altogether, these findings demonstrate that the essential oil of *E. pyriformis* exerts anti-inflammatory effects through multiple mechanisms, including the inhibition of lipid-derived mediators and the suppression of pro-inflammatory cytokine expression. Considering its sesquiterpene-rich composition and the concordance between in vitro and in vivo results, *E. pyriformis* emerges as a promising natural source of bioactive compounds for the development of safe and effective anti-inflammatory agents.

## Figures and Tables

**Table 1:** Compounds identified in the essential oil of *E. pyriformis* by GC/MS.

Compounds	RT	AI	AI_calc_	Relative Abundance (%)
δ-elemeno	22.108	1331	1335	2.62
α-ylangene	23.577	1366	1373	0.3
α-copaeno	23.840	1372	1374	1.8
β-bourboneno	24.178	1380	1387	0.93
β-elemeno	24.409	1386	1389	1.72
β-cariofileno	25.664	1416	1417	7.89
β-gurjuneno	26.070	1426	1431	2.37
Aromadendreno	26.448	1435	1439	0.48
6,9-guaiadieno	26.539	1438	1442	0.29
α-humuleno	27.117	1452	1452	1.13
alo-aromadendreno	27.321	1457	1458	1.3
γ-muuroleno	28.172	1478	1478	18.44
δ-selineno	28.609	1489	1492	1.73
Biciclogermacreno	28.763	1493	1500	6.66
α-muuroleno	28.883	1495	1500	3.54
γ-cadineno	29.450	1510	1513	1.52
δ-cadineno	29.665	1516	1522	9.02
Zonareno	29.825	1520	1528	0.51
trans-cadina-1,4-dieno	30.210	1530	1533	0.47
α-cadineno	30.381	1534	1537	0.67
α-calacoreno	30.567	1539	1544	0.53
Germacreno B	31.232	1556	1559	3.46
Espatulenol	31.954	1575	1577	2.48
Caryophyllene oxyde	32.135	1579	1582	0.51
Globulol	32.299	1584	1590	2.62
Guaiol	32.639	1592	1600	2.26
Eremoligenol	33.816	1624	1629	1.52
Iso-espatulenol	34.104	1632	1623	1.32
epi-α-cadinol	34.410	1640	1638	1.25
α-muurolol	34.579	1645	1644	1.32
α-cadinol	34.905	1654	1652	5.1
Neo-intermedeol	35.005	1657	1658	0.66
Eudesma-4(15).7-dien-1β-ol	36.197	1689	1687	0.61
Total				87.03
Sesquiterpene hydrocarbons				67.38
Oxygenated sesquiterpene				19.65

RT: Retention time; AI: Literature Arithmetic Index; AI_calc_: Calculated Arithmetic Index.

## Data Availability

The original contributions presented in this study are included in the article/Supplementary Material. Further inquiries can be directed to the corresponding author.

## Supplementary Materials

The following supporting information can be downloaded at: https://www.mdpi.com/article/10.3390/molecules31020342/s1, Figure S1: Antiedematogenic effect of the essential oil from *Eugenia pyriformis* (EP) on carrageenan-induced paw edema in mice. Figure S2: Antiedematogenic effect of the essential oil from *Eugenia pyriformis* (EP) on Prostaglandin E_2_ paw edema in mice. Figure S3: Antiedematogenic effect of the essential oil from *Eugenia pyriformis* (EP) on Bradykinin paw edema in mice. Figure S4: Antiedematogenic effect of the essential oil from *Eugenia pyriformis* (EP) on Compound 48/80 paw edema in mice.
