# Supplementary material for: Chemical Profiling and Multimodal Anti-Inflammatory Activity of Eugenia pyriformis Leaves Essential Oil
Source: Molecules. 2026 Jan 19;31(2):342. doi: 10.3390/molecules31020342 (PMC12844175; doi:10.3390/molecules31020342)
Supplement: Supplementary file 1 [file molecules-31-00342-s001.zip › molecules-4074777-supplementary.pdf]

Supplementary material

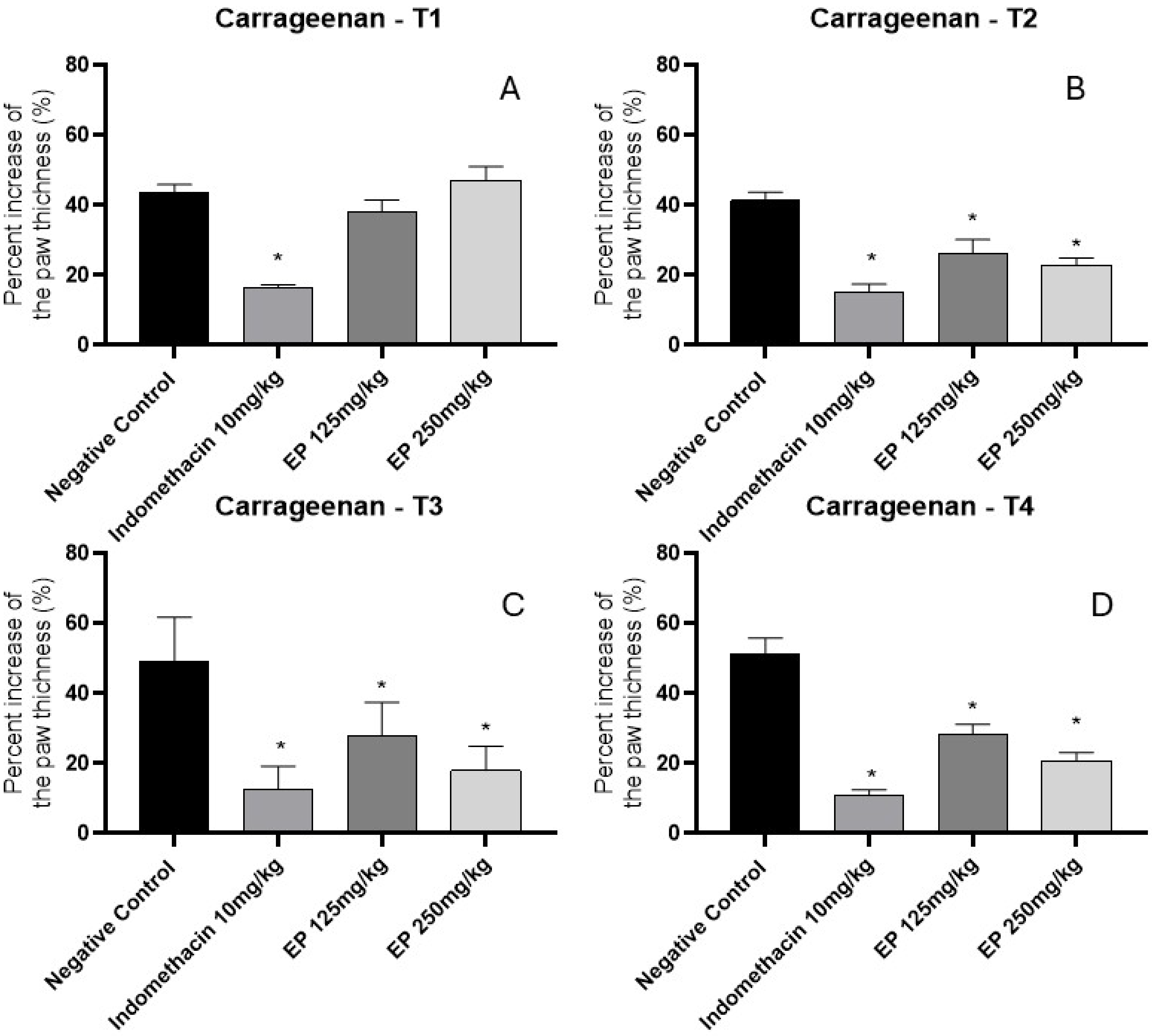

S1: Antiedematogenic effect of the essential oil from *Eugenia pyriformis* (EP) on carrageenan-induced paw edema in mice. Edema formation was evaluated as the percentage increase in paw thickness at different time points after carrageenan

injection: (A) T1, (B) T2, (C) T3, and (D) T4. Animals were treated with EP (125 or 250 mg/kg), indomethacin (10 mg/kg, positive control), or vehicle (negative control). Results are expressed as mean ± standard deviation (SD). $p < 0.05$ compared to the negative control group (one-way ANOVA followed by Dunnett's multiple comparisons test. P-values < 0.05 were considered significant.

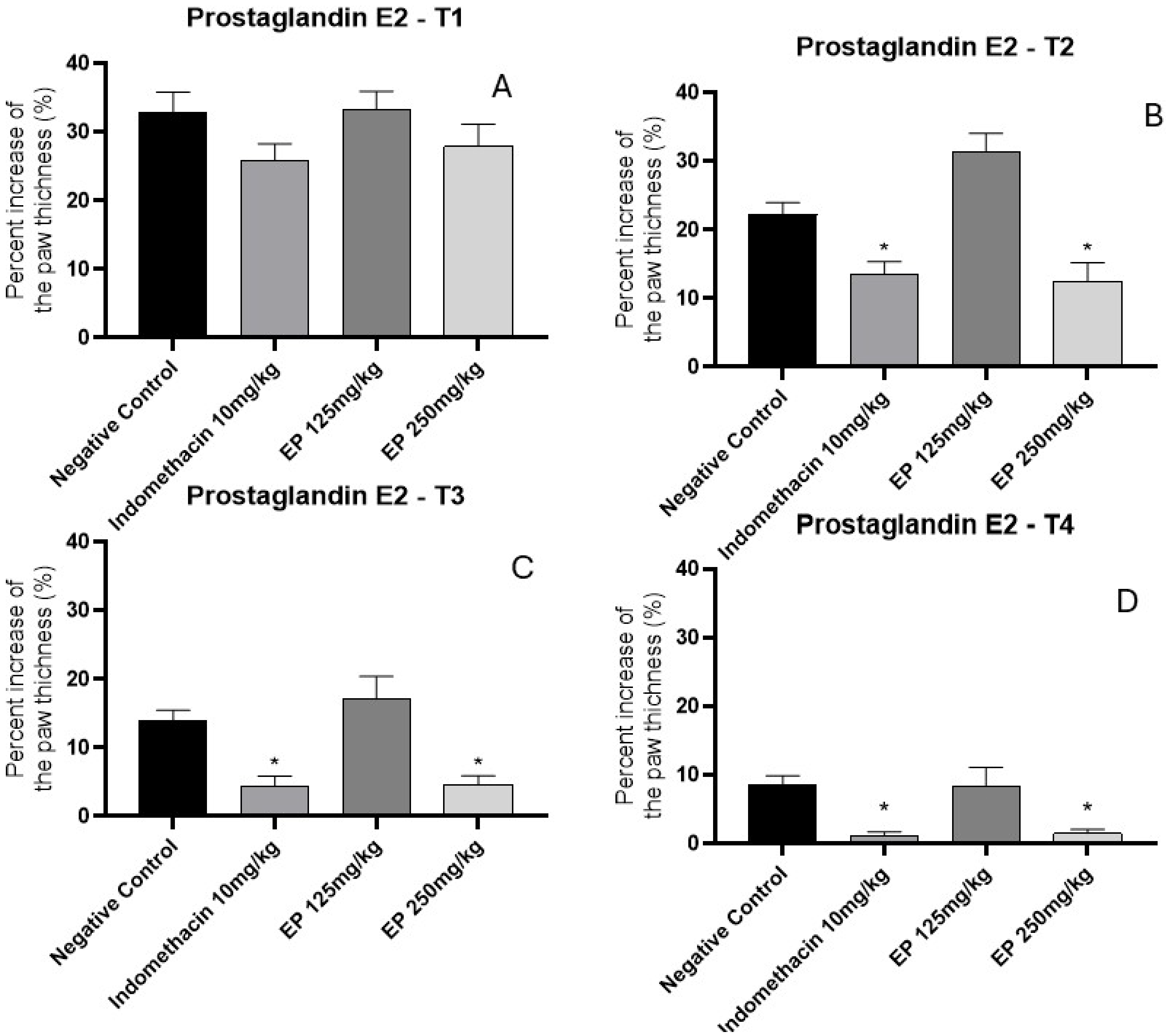

S2:Antiedematogenic effect of the essential oil from *Eugenia pyriformis* (EP) on Prostaglandin E_2 paw edema in mice. Edema formation was evaluated as the percentage increase in paw thickness at different time points after carrageenan injection: (A) T1, (B) T2, (C) T3, and (D) T4. Animals were treated with EP (125 or 250 mg/kg), indomethacin (10 mg/kg, positive control), or vehicle (negative control). Results are expressed as mean ± standard deviation (SD). $p < 0.05$ compared to the negative control group (one-way ANOVA followed by Dunnett's multiple comparisons test. P-values < 0.05 were considered significant.

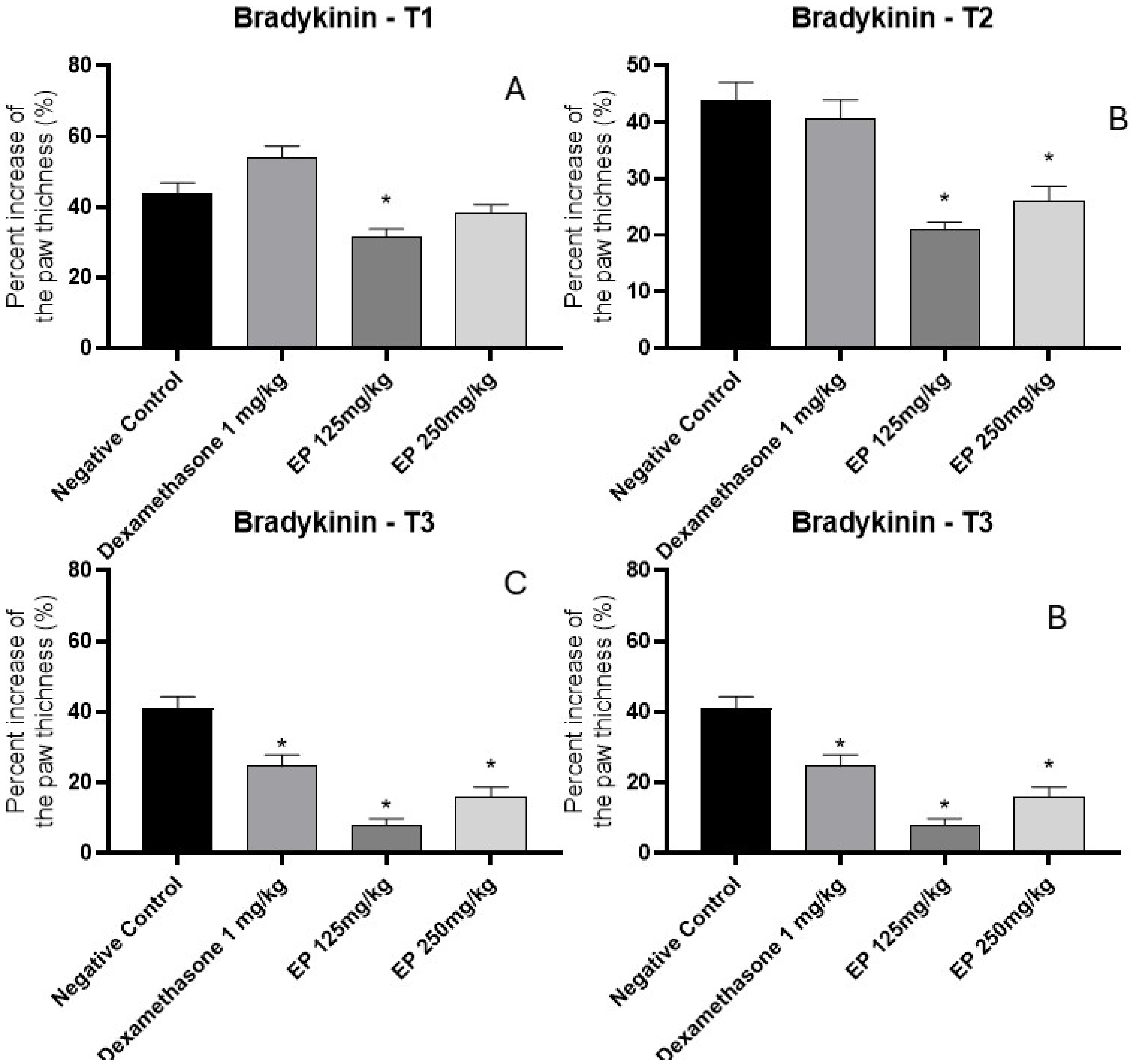

S3: Antiedematogenic effect of the essential oil from *Eugenia pyriformis* (EP) on Bradykinin paw edema in mice. Edema formation was evaluated as the percentage increase in paw thickness at different time points after carrageenan injection: (A) T1, (B) T2, (C) T3, and (D) T4. Animals were treated with EP (125 or 250 mg/kg), indomethacin (10 mg/kg, positive control), or vehicle (negative control). Results are

expressed as mean ± standard deviation (SD). $p < 0.05$ compared to the negative control group (one-way ANOVA followed by Dunnett's multiple comparisons test. P-values < 0.05 were considered significant.

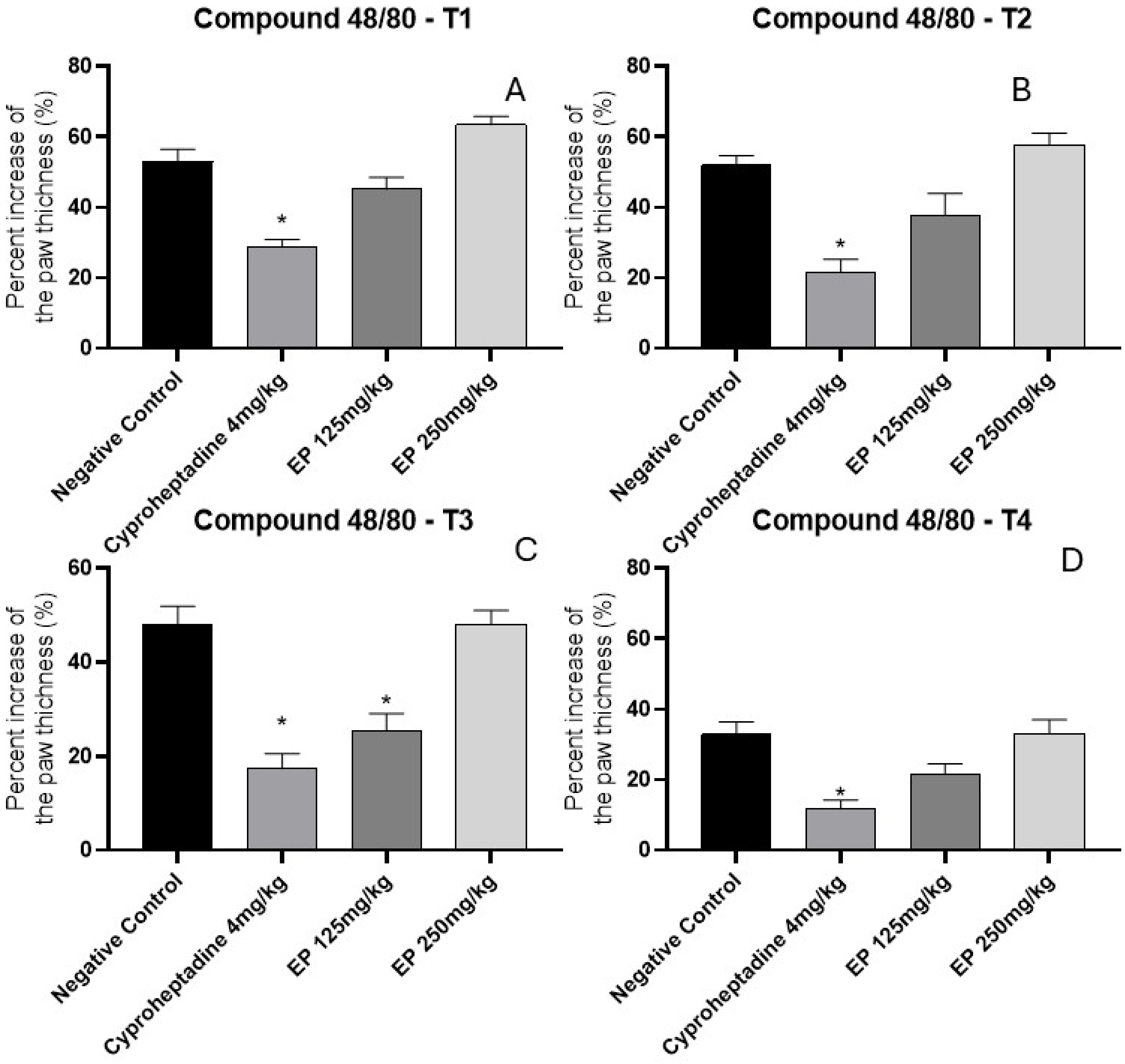

S4: Antiedematogenic effect of the essential oil from *Eugenia pyriformis* (EP) on Compound 48/80 paw edema in mice. Edema formation was evaluated as the percentage increase in paw thickness at different time points after carrageenan injection: (A) T1, (B) T2, (C) T3, and (D) T4. Animals were treated with EP (125 or 250 mg/kg), indomethacin (10 mg/kg, positive control), or vehicle (negative control). Results are expressed as mean ± standard deviation (SD). $p < 0.05$ compared to the negative control group (one-way ANOVA followed by Dunnett's multiple comparisons test. P-values < 0.05 were considered significant.
